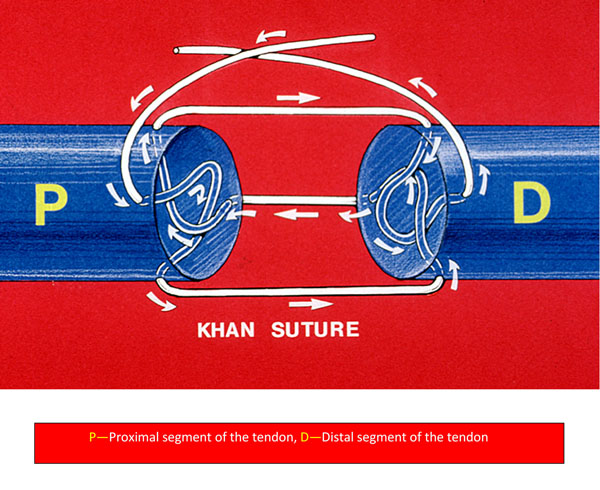# Optimal Zone II flexor tenorraphy

**DOI:** 10.1186/1753-6561-9-S3-A67

**Published:** 2015-05-19

**Authors:** M Ibrahim Khan

**Affiliations:** 1 Department of Plastic Surgery, DHQ Hospital, Rawalpindi, Pakistan

## 

“There is a great difference between knowing and understanding: You can know a lot about something and not really understand it.â€�. This quote attributed to Charles Kettering explains why the more we know about zone 2 repairs; the less we seem to understand. In this presentation, I will show that our lack of understanding is due entirely to biomechanical gaps in the current knowledge—gaps large enough to drive a truck through.

Flexor tendon groupthink is turning a blind eye to these gaps partly because of their obsession with the blood supply of the flexor tendon and partly because of the control of their zone 2 vocabulary by the ultimate tensile strength of the repair which has blinded them to the proverbial elephant in the room – dorsal gapping at the repair site and its consequences such as adhesions including PIP flexion contractures and ruptures. This presentation will be all about identifying and closing these gaps. So, the biomechanical sine qua nons of an optimal suture for zone 2 flexor tenorrhaphy are:

(1) Non-end placement for optimal loading: State of the art palmer end-placement of the ‘core’ suture and controlled mobilization is a classic case study in unintended biomechanical consequences or U.B.C. Exhibit A is eccentric loading of the flexor tendon, which boomerangs back on the repair site as a negative bending moment with the bending tensile stresses on the dorsum and bending compressive stresses on the palmar side, ripping the repair apart dorsally, resulting in dorsal gap which is exhibit B. This dorsal gap induces instability at the repair site which in turn seduces the tendon sheath to invade the tendon all in the name of stability. If the sheath succeeds, we end up with adhesions including P.I.P. flexion contractures but if it fails the negative consequence will be rupture.

What has made the end-placement a case of “out of the frying pan, in to the fire" is the fantasy marriage of this technique and multi strands such as Savage six strand suture and its endless imitations because the powerful one-two punch of palmar end placement and multi strands does not give the suture just a bite on the palmer side but a savage bite, opening the repair dorsally like a book. Since dorsal gap is initiated by the palmar end-placement of the core suture and propagated by controlled mobilization, the question on every one’s mind is: Can the end-placement be made harmless? Theoretically, if the suture can be placed along the centroid of the flexor tendon, then that will take care of the bending moment and make the end-placement harmless. But that’s impractical because the centroid of flexor tendon is a shifting tech tonic plate between fully extended and fully flexed positions of the finger. So, faced with zone 2 repairs’ technical dilemma of either chasing an elusive centroid or making the centroid virtual, it’s a no brainer that we have to make it virtual and that means ‘non-end placement’.

(2) Large central loops for optimal load bearing: The amount of load that a suture can bear is directly proportional to the frictional resistance at suture-tendon interface which in turn is dependent on: (a) the size of suture material (b) the size of loops the suture makes in the tendon

(Suture anchoring). But the standardized size of the suture for Zone 2 repairs leaves us with one and only one choice to enhance the load bearing capability of the suture and that is to enlarge the size of the suture loops. So, quite contrary to the current state of the art practice, what is needed is utilization of large loops in the center of the tendon with multi planner obliquities but no locking for enhanced anchoring.

(3) Multi strands for optimal tensile strength: A multi strand suture can and will multiply the tensile strength of its strands provided that there’s simultaneous loading of suture strands of similar stiffness. The exclusive prerequisites for this multiplication are:

(a) All strands must share the same suture with knot in one strand, to ensure simultaneous loading.

(b) All strands must be see-sawing (to and fro), to ensure even strand tension.

(c) All strands must deploy similar loops, to ensure even strand stiffness.

The fact that the flexor tendon groupthink is turning a blind eye to the mechanical realities of multistrands will be made crystal clear in the logical and factual analysis of these so called

‘Multistrands’.

So, what is needed isn’t another strong suture but a smart suture like the smart phone.

My suture, shown below, embodies the above stated biological and mechanical attributes and therefore has the most compelling biomechanical incentive: the prevention of dorsal gap with all its attendant consequences and in so doing; it solves the unresolved issues of adhesions including P.I.P. flexion contractures and ruptures. It also nips the technical anxiety of zone 2 repairs in the bud by preventing inversion and eversion of the tendon ends as it uses the epitenon-first technique.

**Figure 1:**